# Rapid ^18^F-FDG Uptake in Brain of Awake, Behaving Rat and Anesthetized Chicken has Implications for Behavioral PET Studies in Species With High Metabolisms

**DOI:** 10.3389/fnbeh.2018.00115

**Published:** 2018-06-05

**Authors:** Maria E. L. Gold, Mark A. Norell, Michael Budassi, Paul Vaska, Daniela Schulz

**Affiliations:** ^1^Division of Paleontology, American Museum of Natural History, New York, NY, United States; ^2^Department of Anatomical Sciences, Stony Brook University, Stony Brook, NY, United States; ^3^Department of Biology, Suffolk University, Boston, MA, United States; ^4^Department of Biomedical Engineering, Stony Brook University, Stony Brook, NY, United States; ^5^Department of Radiology, Stony Brook University, Stony Brook, NY, United States; ^6^Biosciences Department, Brookhaven National Laboratory, Upton, NY, United States; ^7^Department of Psychology, Yeditepe University, Istanbul, Turkey

**Keywords:** PET, RatCAP, mammal, avian, model species, metabolism

## Abstract

Brain-behavior studies using ^18^F-FDG PET aim to reveal brain regions that become active during behavior. In standard protocols, ^18^F-FDG is injected, the behavior is executed during 30–60 min of tracer uptake, and then the animal is anesthetized and scanned. Hence, the uptake of ^18^F-FDG is not itself observed and could, in fact, be complete in very little time. This has implications for behavioral studies because uptake is assumed to reflect concurrent behavior. Here, we utilized a new, miniature PET scanner termed RatCAP to measure uptake simultaneously with behavior. We employed a novel injection protocol in which we administered ^18^F-FDG (i.v.) four times over two 2 h to allow for repeated measurements and the correlation of changes in uptake and behavioral activity. Furthermore, using standard PET methods, we explored the effects of injection route on uptake time in chickens, a model for avians, for which PET studies are just beginning. We found that in the awake, behaving rat most of the ^18^F-FDG uptake occurred within minutes and overlapped to a large extent with ^18^F-FDG data taken from longer uptake periods. By contrast, behavior which occurred within minutes of the ^18^F-FDG infusion differed markedly from the behavior that occurred during later uptake periods. Accordingly, we found that changes in ^18^F-FDG uptake in the striatum, motor cortex and cerebellum relative to different reference regions significantly predicted changes in behavioral activity during the scan, if the time bins used for correlation were near the injection times of ^18^F-FDG. However, when morphine was also injected during the scan, which completely abolished behavioral activity for over 50 min, a large proportion of the variance in behavioral activity was also explained by the uptake data from the entire scan. In anesthetized chickens, tracer uptake was complete in about 80 min with s.c. injection, but 8 min with i.v. injection. In conclusion, uptake time needs to be taken into account to more accurately correlate PET and behavioral data in mammals and avians. Additionally, RatCAP together with multiple, successive injections of ^18^F-FDG may be useful to explore changes in uptake over time in relation to changes in behavior.

## Introduction

Positron emission tomography (PET), in conjunction with the glucose-analog ^18^F-fluorodeoxyglucose (^18^F-FDG), has been used to study brain glucose metabolism in relation to behavior (Kornblum et al., 2000; Myers, 2001; Myers and Hume, 2002; Schiffer et al., 2007). Traditionally, these studies entail the injection of ^18^F-FDG, production of behavior, anesthesia, and subsequent scanning. However, the development of a new, miniature, portable PET scanner, termed RatCAP, has made scanning animals without anesthesia possible (Schulz et al., 2011). This scanner attaches to the animal's head and is supported by a counter-weighted pendular structure which provides the animal with considerable freedom of motion during the scan (Schulz et al., 2011). The opportunity to simultaneously measure PET activity and behavior can help clarify the correlation between the two data sets.

In PET imaging, uptake time is traditionally measured in dynamic scans in which radioactivity concentrations within brain regions of interest (ROIs) are tracked over time. Early on, ^18^F-FDG uptake reflects both free tracer in brain tissue and ^18^F-FDG-6-phosphate, the phosphorylated form of ^18^F-FDG that becomes trapped inside the brain cell and indicates glucose consumption (Sokoloff et al., 1977; Gallagher et al., 1978; Reivich et al., 1979). But from the first min of uptake up until 2 h following an intravenous (i.v.) injection, virtually all radioactivity in the brain of mice is in the form of ^18^F-FDG-6-phosphate (Gallagher et al., 1978). Additionally, ^18^F is mostly cleared from arterial blood, a proxy for free tracer in brain tissue, in less than a minute (Schiffer et al., 2007). This implies that the trapping process that may relate to behavior is potentially complete in a minimum of time. Regardless, PET-behavior studies traditionally allow for 30–60 min of tracer uptake or “trapping” in the awake, behaving state before the animal is anesthetized and scanned (Kornblum et al., 2000; Mirrione et al., 2007; Jang et al., 2009; Shackman et al., 2013; Thompson et al., 2014). The PET data resulting from the images are then correlated with behavior that occurred during “tracer uptake.” Because phosphorylated tracer cannot leave the cell until radioactive decay (half-life = 109.7 min), its concentration is relatively stable over time (Gallagher et al., 1978; Schmidt et al., 1996). Thus, the delay from tracer injection to PET imaging an hour later is not problematic for obtaining valid PET data. However, if uptake is actually occurring much faster than the assumed duration of 30–60 min, but 30–60 min worth of behavioral data are correlated with the PET data, then extraneous behavioral data are added or averaged, which could generate misleading results. Hence, uptake time needs to be taken into account to more accurately correlate the PET and behavioral data. In the present study, we have measured uptake time, as defined by the time it takes for the tracer to reach max concentration in the brain of an awake, behaving rat, using RatCAP technology. Moreover, instead of using four rats, we have tested four repetitions in one rat. This repeated-measures approach not only provided confidence in the reliability of our findings, but also allowed for an assessment of transient changes in the brain that might relate to transient changes in the rat's behavior during the PET scan. Thus, the multi-injection protocol might represent a new tool to be used in brain-behavior studies that employ the RatCAP.

Some aspects of biology can affect metabolism and therefore uptake time. An individual animal's metabolism varies depending on its activity, ambient temperature, latitude, food availability, organ, and muscle mass, body size, sex, and age (Mueller and Diamond, 2001; Glazier, 2005; Raichlen et al., 2010; McMurray et al., 2014). In controlled experiments, many of these factors do not significantly vary and so produce no effect. Yet, factors such as behavior, anesthesia, and injection route may affect metabolic rate or the speed at which the tracer reaches the brain and therefore uptake time. These factors have not been studied systematically in all species. Chickens are model animals for birds and non-avian dinosaurs because they are among the more basal extant birds and their biology is well-known (Wagner and Gauthier, 1999; Prum and Dyck, 2003; Welten et al., 2005). Mammals and birds are comparable because of their highly encephalized brains, convergent ecological niches, and high metabolic rates, although differences in glucose metabolism exist between the species. For example, birds have higher levels of glucose in plasma, and the glucose concentration inside the cell is much lower than in plasma whereas it is similar in the mammal (Stevens, 1996). Thus, differences in the uptake of ^18^F-FDG could be expected between rats and chickens. To date, very few ^18^F-FDG studies have been conducted in avians. Parrots and eagles have been imaged for diagnostic veterinary purposes (Souza et al., 2010; Jones et al., 2013), crows to study facial recognition (Marzluff et al., 2012; Cross et al., 2013) and starlings to study flight (Gold et al., 2016). In this proof of concept study, we have used chickens to explore the effects of injection route on the uptake of ^18^F-FDG in forebrain and cerebellum, which represent major divisions in avian neuroanatomy (Balanoff et al., 2013), during wakefulness and under anesthesia. We have tested intravenous (i.v.) and subcutaneous (s.c.) injections because these are recommended routes of drug delivery in birds (Leipold, 1987). Furthermore, we have tested an awake, behaving rat using the RatCAP scanner together with a multi-injection protocol to study uptake times during behavior and to test a new dynamic-like protocol that exploits the potential of the RatCAP scanner. By injecting ^18^F-FDG four times, ~30 min apart, we had the opportunity to study changes in brain uptake that relate to dynamic changes in behavior during the PET scan. We have tested our methods under baseline conditions and following an intervention with morphine. We chose morphine because of its clearly discernible, biphasic effects on motor behavior (Babbini and Davis, 1972; Di Chiara and Imperato, 1988), which we expected to have an observable influence on the PET and behavioral data. We have focused hereby on analyzing ROIs that might link the behavioral activity of the rat to the motor systems of the brain, which include the striatum, motor cortex and cerebellum (Berridge and Whishaw, 1992; Sanes and Donoghue, 2000; Doyon et al., 2003; Kishore et al., 2014). Previously, we could show that ^11^C-raclopride binding to dopamine D2 receptors in the striatum predicted the behavioral activity of rats wearing the RatCAP (Schulz et al., 2011), but the limited binding profile of the tracer did not allow for correlations with other brain regions. Together, the present studies expand current knowledge by providing an estimate of uptake time of ^18^F-FDG in two model species and a first demonstration of the relevance of uptake time to behavior in the rat.

## Methods

### Subjects

A female adult Long-Evans (LE) rat and a female and two male specific pathogen-free chickens (*Gallus gallus domesticus*) were purchased from Charles River Laboratories (Wilmington, MA). The animals were housed in groups until surgery and individually thereafter. The rat was housed in a standard polycarbonate cage. The chickens were kept in 60cm high × 1.5m long × 1.2m wide pens. The animals weremaintained on a 12:12 light:dark cycle (lights on at 7:00 a.m.) with free access to food and water. All procedures were approved by the Institutional Animal Care and Use Committees of Brookhaven National Laboratory, Stony Brook University and the AmericanMuseum of Natural History, and were conducted in accordance with the *Guide for the Care and Use of Laboratory Animals*.

### Surgeries

Details of the surgical procedures were published in Schulz et al. (2011). In short, jugular vein catheters were implanted for infusion of the radiotracer ^18^F-FDG. The same procedures were used in the chickens and rat, except that chickens were anesthetized with a ketamine/xylazinemixture (30:3) injected intramuscularly (i.m.), whereas the rat received ketamine/xylazine (10:1) intraperitoneally (i.p). The rat weighed 360 g at the time of surgery. The chickens varied in age (Table 1). The jugular vein catheters consisted of 10cm silastic tubing with one end attached to a backmount cannula connector pedestal (Plastics One, Roanoke, VA). The loose end of the catheter tubing was inserted approximately 2cm into the jugular vein. A silicone bead on the tubing served as an anchor for sutures. The connector pedestal, which had polyestermesh on the base, wasmounted subcutaneously (s.c.) on the back of the animal. To ensure catheter patency, the lines were flushed every 3 days with gentamicin solution (200 μgml^−1^). Head surgery was performed only in the rat for later attachment of the RatCAP scanner. Eight anchor screws were attached to the skull for stability of a dental-cement cap. A 2-octyl-cyanoacrylate based tissue adhesive was applied to the skull to affix two screw sockets (7mm high, 3mm diameter) onto themidline at bregma and lambda, respectively, for attachment of amagnetic catch thatmates with the RatCAP scanner. Dental cement was packed around the sockets except for their openings on the top.

**Table 1:** Chicken information and imaging combinations for eachmicroPET scan.

**Chicken identification number in Figures 9, 10**	**Sex**	**Age (weeks)**	**Approx. Weight (g)**	**Fasting Time (min)**	**Anesthetic**	**InjectionMethod**	**Tracer dose (mCi)**	**Behavioral Conditions**
#2	Male	7	540	60	Isoflurane	S.C.	1.44	Anesthetized
#2	Male	18	1,320	At least 45	Isoflurane	I.V.	1.43	Awake
#1	Female	27	1,602	300	0.35mL Ket/Xy	I.V.	1.8	Anesthetized
#1	Female	29	1,639	200	0.35mL Ket/Xy	I.V.	1.8	Awake
#3	Male	35	1,690	285	0.35mL Ket/Xy	S.C.	1.6	Awake

### PET imaging

**Figure 1:**
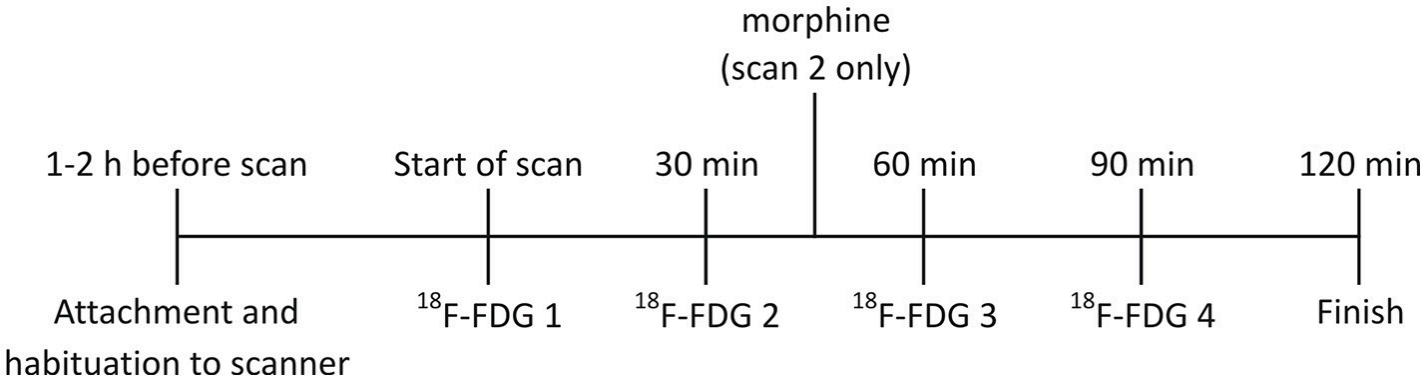
Timeline for RatCAP studies. The rat was attached to the RatCAP 1–2 h before the start of the scan for acclimation to the scanner and the open field. The start of the scan coincided with the first ^18^F-FDG injection. ^18^F-FDG was injected 4 times, approximately 30min apart. In scan 2,morphine (3mg/kg; i.v.) was also administered approximately 15min after the second ^18^F-FDG injection.

**Figure 2:**
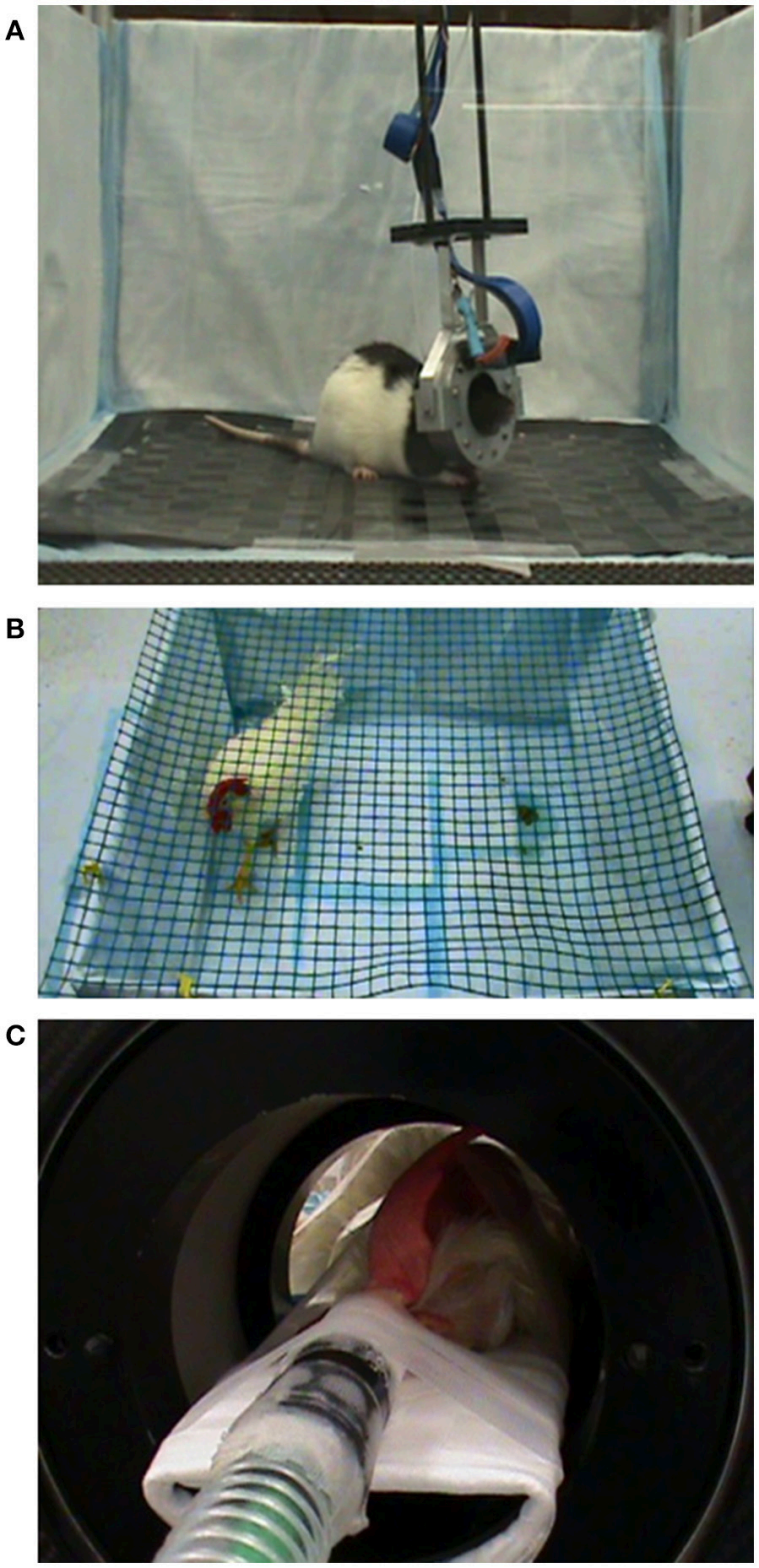
Experimental setup during PET scanning. **(A)** A rat is imaged with the RatCAP scanner while it is awake and spontaneously behaving in an open field. **(B)** A chicken is traversing the pen during uptake of ^18^F-FDG. **(C)** A chicken under isoflurane anesthesia during PET scanning in themicroPET.

The rat wasmoved to the scanner room in its home cage and received a food ration of 50% *ad lib* the night before imaging. It was attached to the RatCAP scanner 1–2 h before the start of the PET scan (see Figure 1 for study timeline). To this end, the animal wasmomentarily anesthetized with a bolus of isoflurane gas. Amagnetic catch was attached to the screw sockets and the animal's head was placed inside the ring of the scanner, allowing the catch to snap onto a piece on the scanner. A 200-cm-long infusion linemade of PE50 tubing was attached to the cannula pedestal on the back of the animal for intravenous (i.v.) administration of the radiotracer. The rat was allowed to wake up and kept in a 50 × 50cm open field with 30cm high walls for the duration of the scan (Figure 2A). The attachment of the scanner to the rat's head eliminatesmotion between the head and scanner and, thus,motion artifacts, and with the help of a pendular support structure allows the rat tomove around and be active during the scan (Schulz et al., 2011). We conducted two PET scans 19 days apart. In both scans, ^18^F-FDG (Cardinal Health, Dublin, OH) was administered as a bolus four times ~30min apart. The start of the scan coincided with the first injection of ^18^F-FDG. Injected doses ranged between 300 and 400 μCi. Each dose was given in 200–300 μl of vehicle as permanufacturer and diluted in 0.9% saline to achieve the desired dose of radiation. In the second scan, we also infused (i.v.) 3mg/kg ofmorphine diluted in saline (1ml/kg) using the same PE50 tubing as the one used for the radiotracer. The infusion took place approximately 15min following the start of the second infusion of ^18^F-FDG. Following each scan, the RatCAP andmagnetic catch were immediately dismounted without the use of anesthesia, and the rat was returned to its home cage. RatCAP data were collected in singles listmode, continually through all injections and the data weremonitored to ensure integrity. The lower level discriminator (energy) threshold was 350 keV. The behavior of the rat during the PET scans was filmed using a video camera (SONY, DCR-SR40) placed ca. 1.5m away from the open field and analyzed *post-hoc*.

**Figure 3:**
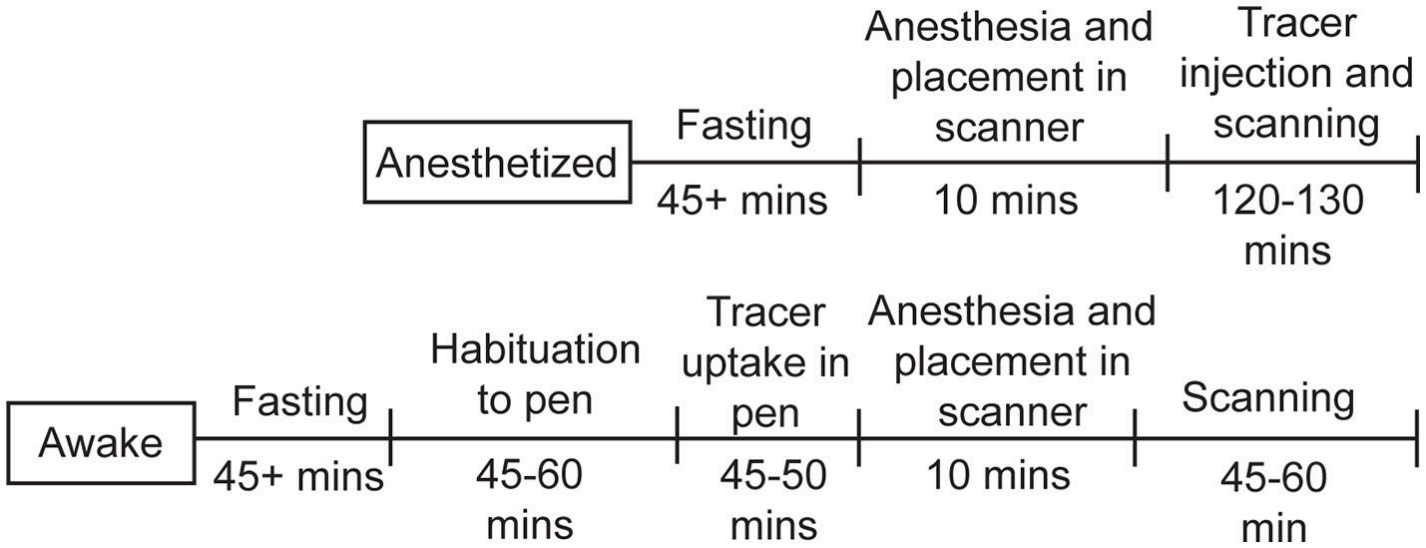
Timeline for chicken studies. For the studies under anesthesia, the chickens were fasted for at least 45min, then anesthetized, injected with ^18^F-FDG, and scanned for up to 130min. For the awake studies, the chickens were fasted for at least 45min, allowed to habituate to the pen, injected with ^18^F-FDG and given time for uptake, then anesthetized and scanned for up to 60min.

The chickens were fasted for at least 45min before the experiments (see Figure 3 for study timeline). In the “awake condition” the chickens were allowed to habituate to a pen (100 × 100cm wide with 45cm high walls) for 45–60min (Figure 2B). Next, they received 1.4–1.8mCi of ^18^F-FDG intravenously (i.v.) via a jugular vein catheter or subcutaneously (s.c.) into a pocket under the right wing near themidpoint of the humerus. The chickens were gently restrained during the injections. They were allowed tomove freely inside the pen for approximately 45min during tracer uptake. The behavior was filmed for later analysis. Anesthesia was then induced with either amixture of oxygen and isoflurane gas (up to 3%) via nose cone or a ketamine/xylazine injection (100mg/ml in a 30:3 ratio; i.m.). Glycopyrrolate (0.3ml; i.m.) was administered to reduce salivation. The animal was fixed on the bed of amicroPET R4 scanner (ConcordeMicrosystems, Knoxville, TN) and the head centered in the field of view (Figure 2C). The respiratory rate ranged between 18 and 36 bpm. Body temperature wasmonitored by a K-type thermometer. A red-light heating lamp was used tomaintain body temperature at 38°C ± 1. In the “anesthetized condition” the start of the PET scan coincided with the injection of the tracer, but the scanning procedures were the same as in the “awake condition.” These scans were conducted over a 7-month period.

### Behavioral analysis

The behaviors were scored using themanual function of EthoVision software (Noldus, Wageningen, NL). In the rat, wemeasured “behavioral activity,” which included all observable forms ofmovement, that is, head turns, forwardmovement,movement of the body withoutmovement of the head, but not the repositioning of the legs while sitting, chewing, andmovements related to breathing and sniffing that were not accompanied by locomotion (Schulz et al., 2011). In chickens, wemeasuredmovements of the body and head, as well as grooming behavior.

### Image analysis

**Figure 4:**
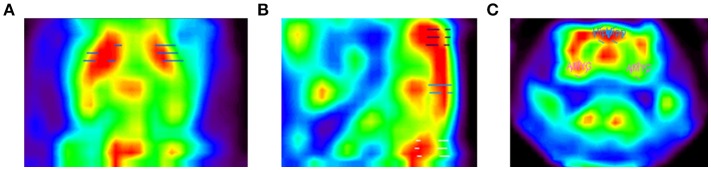
PET images of the rat brain. Depicted are **(A)** a horizontal slice through the brain showing the striatum ROI, **(B)** a sagittal slice showing themotor cortex ROI on top (purple dashes), hippocampus ROI in the center (blue dashes), and cerebellum ROI at the bottom (white dashes), and **(C)** a coronal slice showing the hippocampus (blue circles) and amygdala ROIs (pink circles).

The RatCAP singles listmode data were processed offline to produce prompt and delayed-coincidence (randoms) sinograms with 47 radial and 24 azimuthal bins. The coincidence window (2τ) was 18 ns. Sinograms were reconstructed using theMaximum Likelihood ExpectationMaximization (MLEM) algorithm. The reconstruction included corrections for attenuation, scatter, random coincidences, detector efficiency variations, decay, and deadtime (Southekal et al., 2011). With thesemethods, the spatial resolution of our images is < 2mm FWHM across the field of view (FOV). Each time frame was reconstructed using 100 iterations, and post-smoothing with a 1.8mm FWHM Gaussian kernel was applied. The placement of a given ROI within the coordinate space is shown in Table 2. The ROIs were determined based on slice thickness (1.07mm, AP), the RatCAP FOV (~18mmminus the first and last slice, AP), the first appearance of the striatum (STR), and a rat atlas (Paxinos and Watson, 2007). The ROIs were drawn on the relevant brain areas using ASIPro software (Siemens). For the striatum (STR), we placed a circle shape (4 pixels) on three successive slices both on the left and the right STR (Figure 4A). For themotor cortex (M), a small circle (3 pixels) was placed on the dorsal brain, 1 slice lateral to themidline on either side (Figure 4B). For the hippocampus (HIPP), a small circle (3 pixels) was placed 2 slices ventral from the dorsal surface, immediately to the left and right sides of themidline (Figures 4B,C). For the cerebellum (CB), a circle (4 pixels) was placed in the center of the dorsal brain (Figure 4B). For the amygdala (AMYG), a smaller circle (3 pixels) was placed on two successive slices at the base of the brain, 4–5 slices lateral to themidline on either side, and 4 slices posterior to themost anterior STR (Figure 4C). The amygdala ROI included all nuclei of the amygdala but possibly also portions of the piriform cortex. The data from the left and right sides of all relevant ROIs were averaged for analysis. Because the RatCAP scanner is firmly attached to the screw sockets on the rat's head and the same rat was scanned twice, we used the same ROI template for both scans. Decay-corrected time-activity curves were generated for 1min and 20s time frames.

**Table 2:** Location of ROI in coordinate space.

**Slices in AP plane**	**Cumulativemm**	AP coordinate of start*	AP coordinate of center*	**ROI**	**ROI**
0	1.0766	5.9234	5.3851		
1	2.1532	4.8468	4.3085	M	
2	3.2298	3.7702	3.2319	M	
3	4.3064	2.6936	2.1553	M	STR
4	5.383	1.617	1.0787		STR
5	6.4596	0.5404	0.0021		STR
6	7.5362	−0.5362	−1.0745		
7	8.6128	−1.6128	−2.1511		AMYG
8	9.6894	−2.6894	−3.2277	HIPP	AMYG
9	10.766	−3.766	−4.3043	HIPP	
10	11.8426	−4.8426	−5.3809		
11	12.9192	−5.9192	−6.4575		
12	13.9958	−6.9958	−7.5341		
13	15.0724	−8.0724	−8.6107		
14	16.149	−9.149	−9.6873		
15	17.2256	−10.2256	−10.7639	CB	
16	18.3022	−11.3022	−11.8405	CB	
17	19.3788	−12.3788	−12.9171	CB	
18	20.4554	−13.4554	−13.9937		

* *According to Paxinos and Watson (2007)*.

**Figure 5:**
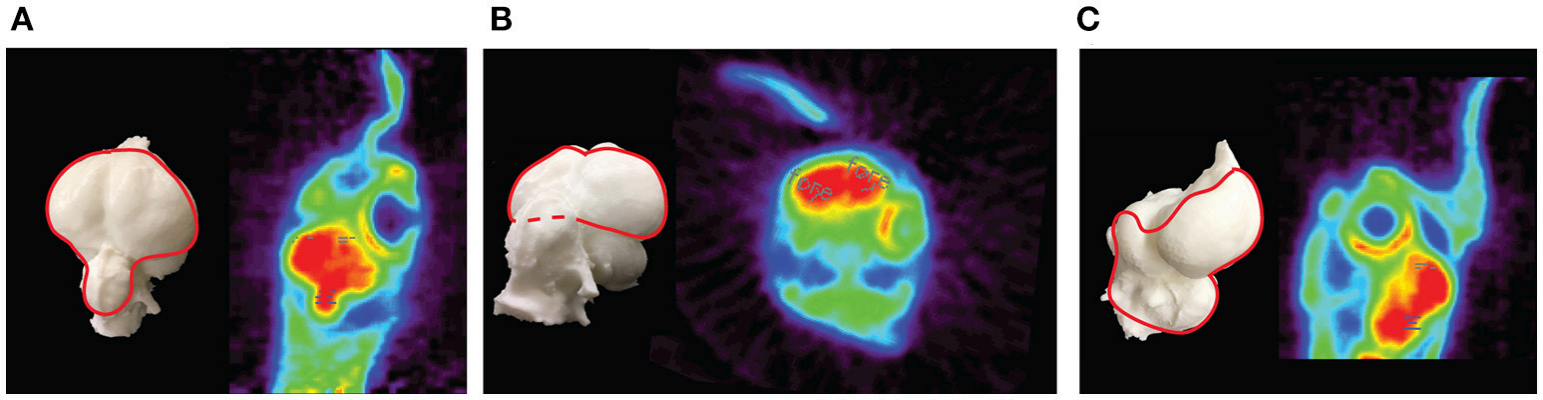
PET images of the chicken brain. Depicted are PET images and corresponding endocasts of the chicken brain. The endocast is a 3-dimensional infilling of the cranial cavity that corresponds to the shape of the brain in a living animal, rotated to be in the same orientation as each PET image. **(A)** A dorsal oblique horizontal slice showing the forebrain (gray dashes) and the cerebellum (blue dashes) ROIs. **(B)** An oblique coronal slice showing the forebrain ROI. **(C)** A sagittal slice showing the forebrain and cerebellum ROIs.

The chicken imaging data were reconstructed using filtered back projection with frequency cutoff at the Nyquist criterion. Using thesemethods, the image slice thickness was 1.21mm (Alexoff et al., 2003). As no digital atlas of a chicken brain is available, ROIs were drawn on two easily visualized divisions of the brain, the forebrain, and cerebellum using ASIPro software (Siemens). In the case of the forebrain, a circle shape (5 pixels) was placed on three successive slices both on the left and the right forebrain (Figures 5A–C). The cerebellum ROI (5 pixels) was placed on three slices (along themidline) just posterior to where the forebrain hemispheres transition to the cerebellum (Figures 5A,C). The data were binned into 3 × 20 s, 9 × 60 s and *n* × 300 s time frames when the ^18^F-FDG injection coincided with the start of the scan, and into 300 s time frames when scans were begun after the awake uptake period. The data (mean nCi/cc) were normalized to injected dose/g of animal weight.

### Data analysis

To analyze our rat data, we divided the 120-min-long data sets into 5min bins (using 20 s frames), because this resulted in a reasonable number of frames to work with in a first exploration of the data. We averaged the PET data and summed the behavioral data for each 5min bin. To show the rapid uptake of ^18^F-FDG in the individual ROIs, we calculated the average uptake that occurred within the first 5min in % of the average uptake that occurred within 25min of every ^18^F-FDG injection. To show the progression of uptake across time, we have also calculated the average uptake in the second 5min, third 5min, fourth 5min and fifth 5min in % of the average uptake across 25min following every injection. Similar to the PET data analyses, the behavioral activity of the rat was analyzed across successive 5min time bins in % of the total activity during 25min following each injection of ^18^F-FDG. To perform PET-behavior correlations, first we took the ratio of ROI_X_/ROI_Y_ for every 5min bin to account for residual ^18^F-FDG from previous injections, where ROI_X_ was either STR,M or CB, and ROI_Y_ was a reference region, that is, either a single ROI or a compound ROI, such as the average ofM and CB. We have tested various reference regions to study the consistency of results across these regions. To analyze the change in uptake of ^18^F-FDG in ROI_X_ relative to ROI_Y_ across time and to correlate that change with the change in behavioral activity, we took the differences between each of two successive 5min time bins in line with our previous study (Schulz et al., 2011). We employed the Spearman rank-order correlation to correlate the changes in uptake with the changes in behavioral activity. We performed these correlations for the differences between all 5min time bins (*n* = 23) as well as the 5min bins closest to the time of the ^18^F-FDG injections (*n* = 8). Always three 5min bins contributed to the difference scores closest to the ^18^F-FDG injections, the bin preceding the injection and the next two bins, which were then subtracted from each other (bin 2minus bin 1 and bin 3minus bin 2), resulting in two difference scores per injection. The injection either coincided with the start of bin 2 or occurred at some point during bin 2. Because the first ^18^F-FDG injection coincided with the start of the scan and, thus, a bin preceding the injection didn't exist, all three 5min bins that contributed to the difference scores followed the first injection of ^18^F-FDG. In correlation analysis, we tested linear functions for significance using the Spearman correlation, but also fitted quadratic and cubic functions to the data in order to detect curvilinear relationships.

## Results

### PET-behavior studies in rat using ratCAP

**Figure 6:**
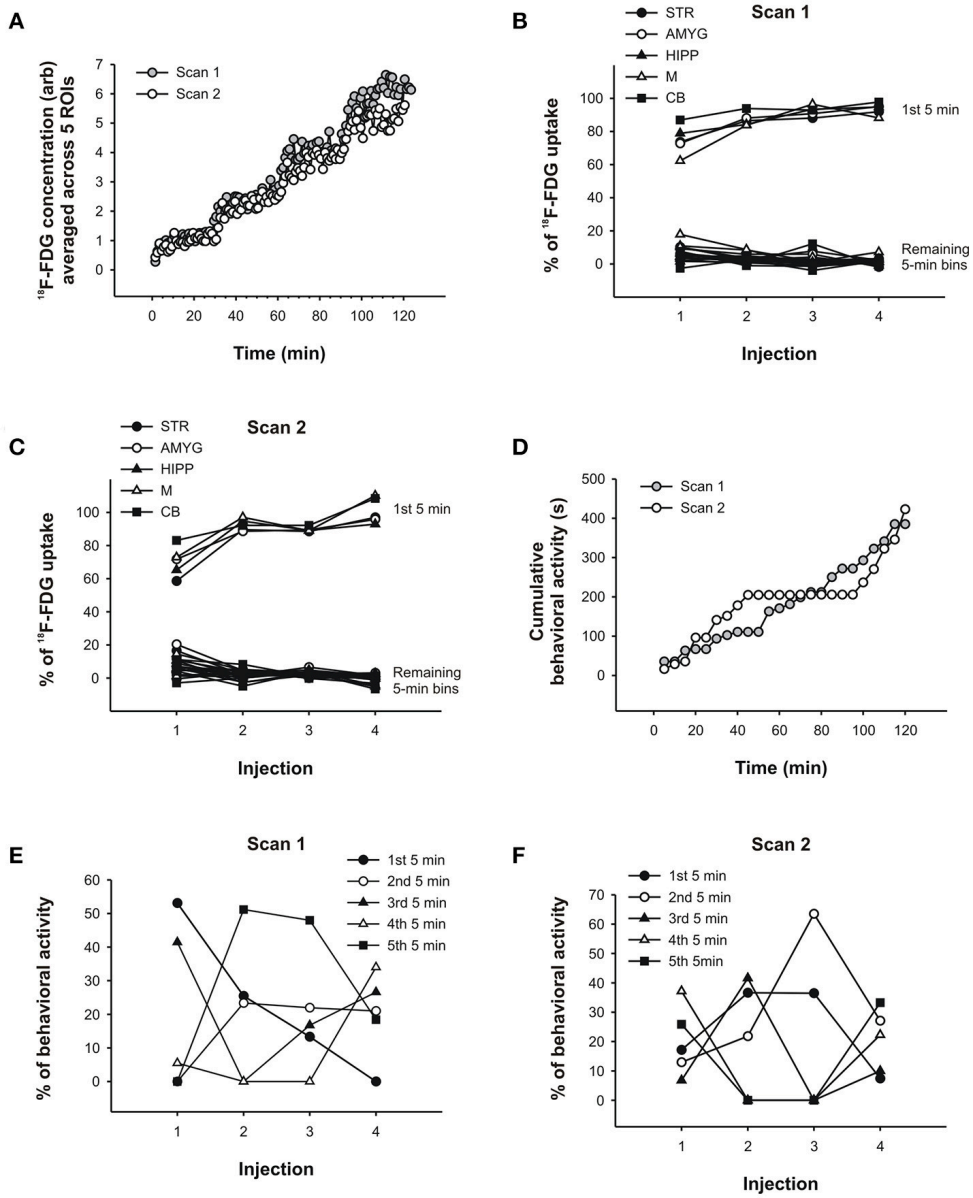
PET data and behavioral analysis for the rat. **(A)** Time-activity curves of scan 1 and scan 2 averaged across 5 ROIs, respectively. ^18^F-FDG uptake in **(B)** scan 1 and **(C)** scan 2 across successive 5min time bins in % of the total uptake during 25min following each injection of ^18^F-FDG.Most of the uptake occurred within the first 5min following each injection. These results were similar for all 5 brain regions examined. **(D)** Behavioral activity (s) was summed into 5min time bins and cumulated, that is, each successive 5min bin was added to the previous sum. In this way, a slope in the behavioral response curve indicates that the rat displayed activity, whereas flat regions indicate the absence of activity. The prolonged absence of activity starting at 45min in scan 2 coincides with the injection ofmorphine. Similar to the PET data analyses in **(B,C)**, the behavioral activity of the rat in scan 1 **(E)** and scan 2 **(F)** was analyzed across successive 5min time bins in % of the total activity during 25min following each injection of ^18^F-FDG. The behavior was distributed across time in fairly unpredictable ways. STR, striatum; AMYG, amygdala; HIPP, hippocampus;M,motor cortex; CB, cerebellum.

To show the uptake of ^18^F-FDG in the behaving rat, we constructed time-activity curves for both scans consisting of 1min time frames that were averaged for STR, AMYG, HIPP,M, and CB. The scans lasted over 2 h and included four ^18^F-FDG injections, as indicated by the sudden increases in uptake (Figure 6A). It can be seen thatmost of the tracer was absorbed within the first fewmin of injection after which uptake appeared to reach a plateau. In fact, when we calculated uptake for each 5min period in % of the first 25min following each injection, we found thatmost of the tracer was taken up in the first 5min and that very little information was added after that. For all ROIs averaged, 75% of the uptake in scan 1 occurred in the first 5min following the first injection, and 87, 92, and 94% were taken up in the first 5min following the second, third and fourth injections, respectively (see Figure 6B for data from individual ROIs). Similarly, whenmorphine was also injected during the scan, 70, 92, 90, and 100% of uptake occurred, on average for all ROIs, in the first 5min following the first, second, third, and fourth injections of ^18^F-FDG, respectively (see Figure 6C for data from individual ROIs).

Thus, while the PET data appear to be similar for the first 5min and 25min following each ^18^F-FDG injection, the behavior changedmarkedly across time. To show the levels of behavioral activity across scan time, we summed the activity into 5min bins and cumulated the bins, so that the display of behavioral activity is represented by a slope increase and the absence of activity by the flat regions in the behavioral response curves (Figure 6D). In the first scan, the rat showed progressively less behavioral activity across the first 5min bins following each ^18^F-FDG injection (Figure 6E). Apart from this regularity, there was no other. The first 5min of behavioral activity were not predictive of the remaining 5min bins, not in the first scan (Figure 6E) or the second scan (Figure 6F). In scan 1, 53% of the behavioral activity that occurred within the first 5min was also present in the first 25min following the first ^18^F-FDG injection. This percentage dropped to 0% with repeated injections. That is, the activity that the animal displayed within the first 25min occurred at some point past the first 5min, and thus was not at all represented in the first 5min whenmost tracer was taken up in the individual ROIs. Similarly, in the second scan there was an overlap in the data from the first 5min and 25min that ranged between 37 and 7% only. This indicates that different resultsmight be obtained when the PET data are correlated with 5min or 25min worth of behavior, since the behavioral data contained in these time bins are very different from each other. Thus, correlating the first 5min of ^18^F-FDG data with the first 5min of behavioral data (but not longer periods)might reduce the confounding effects of subsequent behaviors when correlating the two data sets.

**Figure 7:**
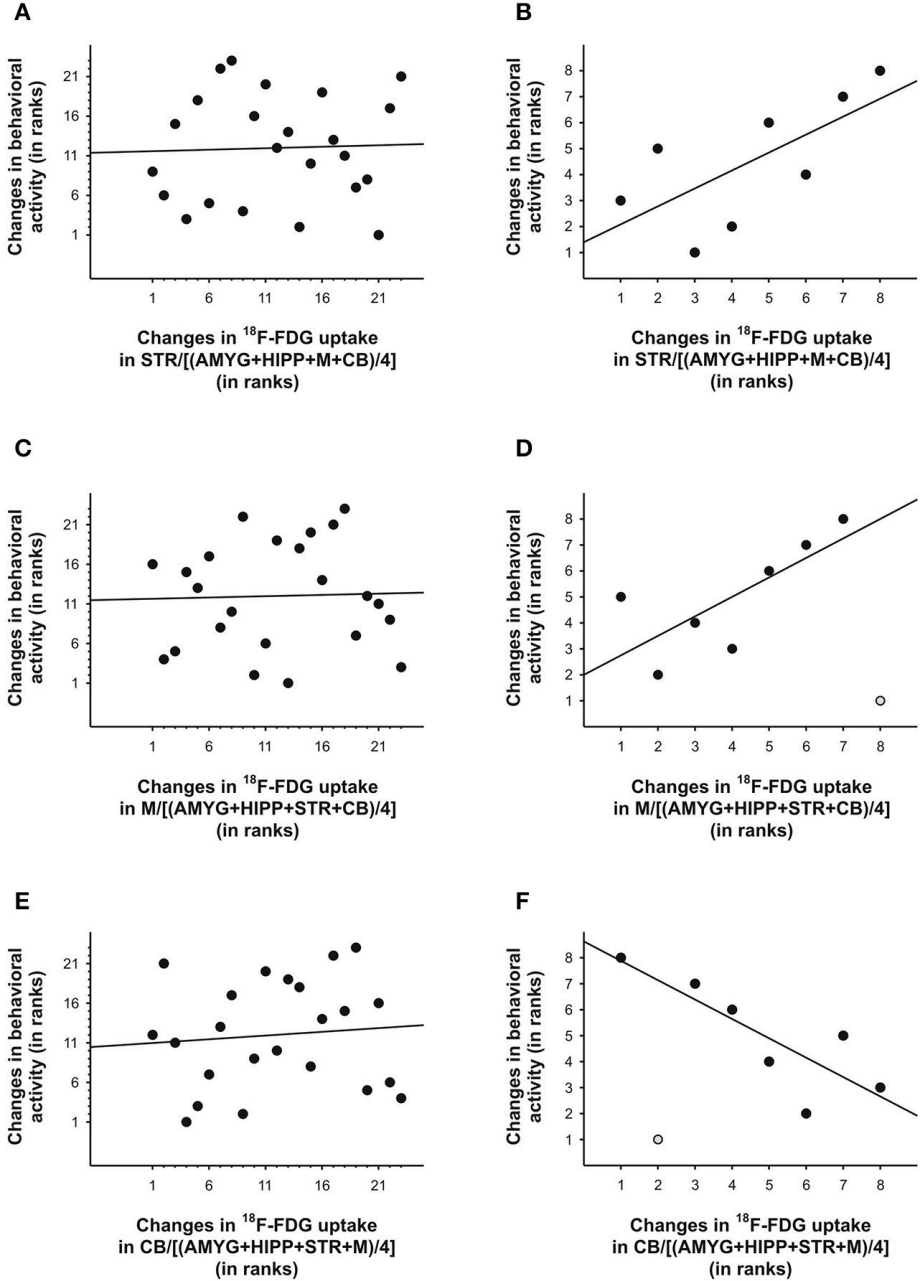
Changes in ^18^F-FDG uptake correlated with changes in behavioral activity across scan 1 in rat. We correlated changes in ^18^F-FDG uptake, i.e., differences between successive 5min time bins, in the **(A,B)** striatum (STR), **(C,D)**motor cortex (M), and **(E,F)** cerebellum (CB) relative to a reference region (the average uptake in a combination of four ROIs) with changes in behavioral activity for the same time frames. When all 5min time frames (*n* = 23) were included for analysis **(A,C,E)**, changes in ^18^F-FDG uptake did not significantly predict changes in behavioral activity across time. By contrast, for the time frames (*n* = 7–8) near the times of the ^18^F-FDG injections **(B, D, F)**, changes in uptake in STR,M, and CB explained a large proportion of the variance in behavioral activity. The data are represented as ranks. Rank 1 indicates the largest decrease, whereas the highest rank indicates the largest increase between any of two successive 5-min-long time frames. When an outlier (light gray) was present **(D, F)**, the regression line was fitted to the remaining data.

To further explore this idea, we correlated the PET and behavioral data using either all 5min time frames from the entire scan or only those time frames that captured the data near the injection times. To this end, we took the ratio of uptake in one ROI over uptake in another ROI to account for residuals from previous injections. Next, we calculated the differences between the 5min time frames on the PET curve and correlated these with the differences in behavioral activity for the same time frames, similar to our approach in our previous study (Schulz et al., 2011). We found that under baseline conditions, changes in uptake in STR,M, and CB relative to various reference regions were predictive of changes in behavioral activity during the scan, but only when the 5min bins near the times of the ^18^F-FDG injections were considered, not when all 5min time bins were included in the analyses (Table 3). We fitted both linear and polynomial functions to the data, and in every single case the explained variance was greater (or far greater) when only the time bins near the times of the injections were included for correlation analysis. For example, the inclusion of all 5min bins did not result in a significant linear correlation between changes in ^18^F-FDG uptake in STR relative to a compound reference region and changes in behavioral activity (*r* = 0.04, *p* = 0.87; Figure 7A), but larger increases in uptake were predictive of larger increases in behavioral activity for the time points near the ^18^F-FDG injections (*r* = 0.69, *p* = 0.06; Figure 7B). Similarly, changes in ^18^F-FDG uptake inM across scan time were not significantly correlated with changes in behavior when all time bins were included for analysis (*r* = 0.03, *p* = 0.88; Figure 7C), but predicted changes in behavior when the time bins near the ^18^F-FDG injections were included (*r* = 0.75, *p* = 0.05; Figure 7D).Moreover, the variability in uptake in CB did not explain a significant proportion of the variance in behavior when all time bins were included (*r* = 0.09, *p* = 0.67; Figure 7E), but explained a large proportion of the variance when the time bins near the ^18^F-FDG injections were included (*r* = −0.82, *p* = 0.02; Figure 7F).

**Table 3:** Changes in uptake in ROI relative to a reference region correlated with changes in behavioral activity across scan 1.

**Predict behavioral activity from**	**Coefficient r**		**Explained variance (%)**			
**ROI ratio**	**Linear**		**Quadratic**		**Cubic**	
	***n* = 23**	***n* = 7-8**	***n* = 23**	***n* = 7-8**	***n* = 23**	***n* = 7-8**
	**All time bins**	**Near injection**	**All time bins**	**Near injection**	**All time bins**	**Near injection**
**STR/**						
AMYG	−0.20 (0.37)	**0.79 (0.02)**	11.62	82.20	21.40	93.89
AMYG+HIPP	0.10 (0.67)	**0.67 (0.07)**	1.50	62.81	9.65	63.39
HIPP	0.29 (0.18)	0.48 (0.23)	12.95	52.66	13.42	52.99
M	0.03 (0.89)	0.60 (0.12)	12.00	36.33	37.77	66.68
M+CB	0.11 (0.63)	**0.76 (0.03)**	6.49	62.64	30.86	63.54
CB	0.03 (0.89)	***0.96 (0.0005)***	7.75	*94.05*	24.51	*96.43*
AMYG+HIPP+M+CB	0.04 (0.87)	**0.69 (0.06)**	1.26	60.43	14.32	60.58
Average *R*^2^ for STR (%)	2.14	51.74	7.65	64.45	21.70	71.07
**M/**						
AMYG	−0.20 (0.37)	*0.32 (0.48)*	6.40	*15.48*	16.42	*20.83*
AMYG+HIPP	−0.03 (0.91)	*0.32 (0.48)*	0.51	*10.37*	9.92	*10.97*
AMYG+HIPP+STR	0.01 (0.96)	*0.18 (0.70)*	0.42	*29.76*	16.32	*77.98*
HIPP	0.22 (0.32)	*0.61 (0.15)*	4.95	*55.61*	27.55	*56.21*
STR+CB	0.001 (0.99)	***0.75 (0.05)***	16.92	*71.6*	17.03	*86.48*
CB	−0.08 (0.73)	***0.89 (0.007)***	11.44	*81.8*	31.42	*82.4*
AMYG+HIPP+STR+CB	0.03 (0.88)	***0.75 (0.05)***	6.61	*78.74*	16.50	*88.27*
Average *R*^2^ forM (%)	1.38	36.09	6.75	49.05	19.31	60.45
**CB/**						
AMYG	0.005 (0.98)	–***0.93 (0.003)***	2.57	*87.76*	19.20	*88.35*
AMYG+HIPP	0.09 (0.67)	–***0.82 (0.02)***	5.32	*77.04*	30.40	*77.64*
HIPP	0.14 (0.52)	–***0.68 (0.09)***	9.22	*49.49*	25.50	*51.87*
STR+M	0.07 (0.74)	–***0.75 (00.05)***	2.88	*82.82*	28.00	*97.7*
AMYG+HIPP+STR+M	0.09 (0.67)	–***0.82 (0.02)***	5.33	*77.04*	31.49	*77.64*
Average *R*^2^ for CB (%)	0.86	64.69	5.06	74.83	26.92	78.64
Average *R*^2^ for all ROIs (%)	1.46	43.92	6.49	62.78	22.64	70.05

*STR, striatum; AMYG, amygdala; HIPP, hippocampus;M,motor cortex; CB, cerebellum. AMYG+HIPP andM+CB, average of two regions; AMYG+HIPP+STR, average of three regions, etc. Results are based on rank-order correlations. Bold, p ≤ 0.09; italic, without outlier. Number in brackets, P-value*.

**Figure 8:**
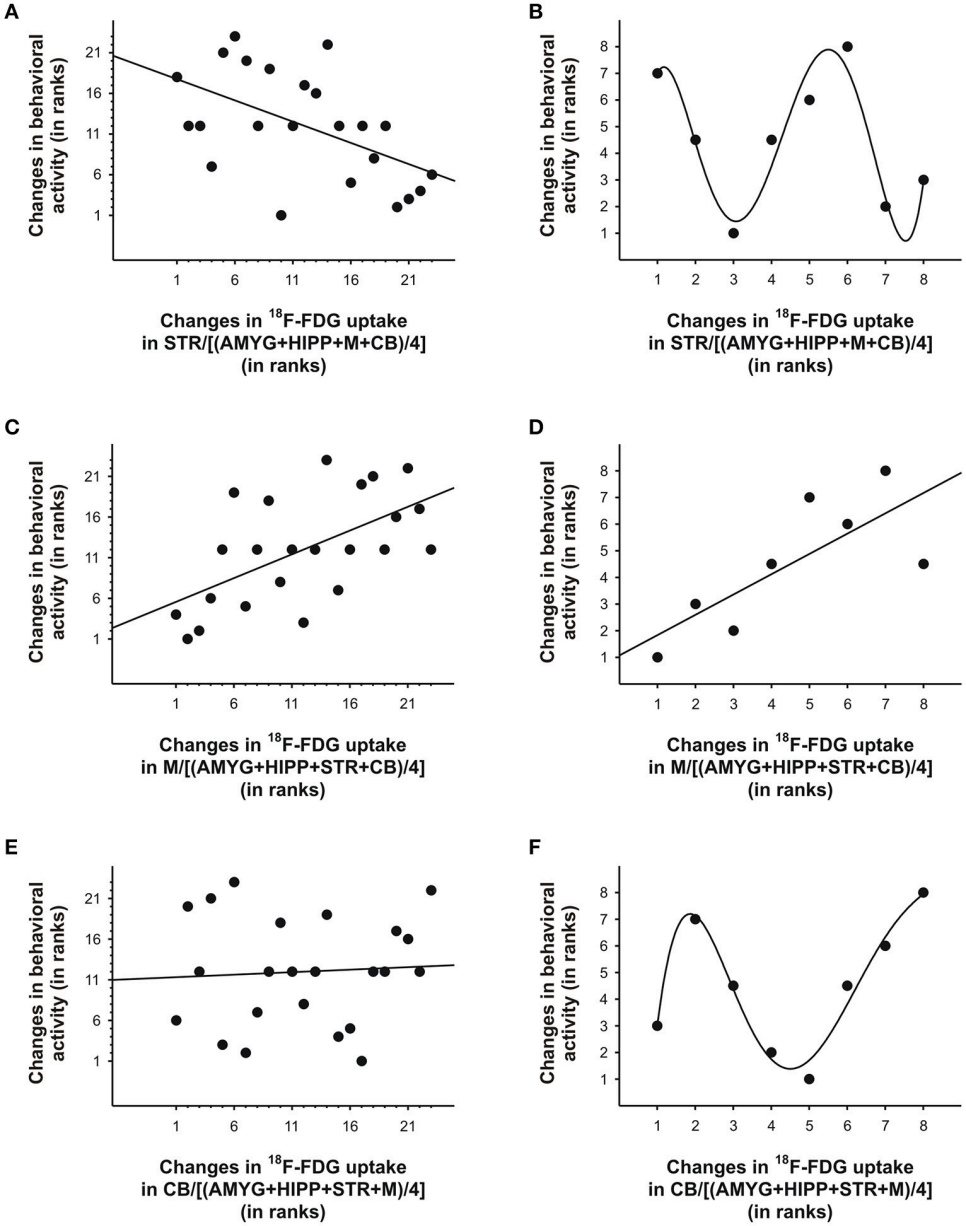
Changes in ^18^F-FDG uptake correlated with changes in behavioral activity across scan 2 in rat. We correlated changes in ^18^F-FDG uptake, i.e., differences between successive 5min time bins, in the **(A, B)** striatum (STR), **(C, D)**motor cortex (M), and **(E, F)** cerebellum (CB) relative to a reference region (the average uptake in a combination of four ROIs) with changes in behavioral activity for the same time frames. When all 5min time frames (*n* = 23) were included for analysis **(A, C, E)**, changes in ^18^F-FDG uptake in the STR andM, but not CB, significantly predicted changes in behavioral activity across time. When only the time frames (*n* = 7-8) near the ^18^F-FDG injections **(B, D, F)** were included for analyses, a linear function could be used to predict changes in behavioral activity from changes in uptake inM **(D)**, but polynomial functions provided the best fit for the data from STR **(B)** and CB **(F)**. For illustration, we fitted a quintic polynomial to the data **(B, F)**. The data are represented as ranks. Rank 1 indicates the largest decrease, whereas the highest rank indicates the largest increase between any of two successive 5-min-long time frames.

In the second scan, in which we tested amorphine intervention, we obtained a somewhat different picture. Although on average, the variance in behavioral activity was still best explained by the variance in uptake in STR,M, and CB when only the 5min bins near the times of the ^18^F-FDG injections were included in the analyses, a large proportion of the variance in behavioral activity was also explained by the uptake data from the entire scan (Table 4). When we fitted a linear regression line to the data, larger increases in STR uptake relative to a compound reference region predicted significantly larger decreases in behavioral activity when all time bins were included in the analysis (*r* = −0.53, *p* = 0.009; Figure 8A), but not when the time bins near the ^18^F-FDG injections were included (*r* = −0.22, *p* = 0.61), as the latter had a curvilinear association with behavior (Figure 8B). Changes inM uptake had positive, linear associations with changes in behavioral activity across the entire scan (*r* = 0.59, *p* = 0.003; Figure 8C) and for the time bins near the ^18^F-FDG injections (*r* = 0.77, *p* = 0.03; Figure 8D). By contrast, changes in CB uptake did not predict a significant proportion of the variability in behavioral activity when all time bins were included for analysis (*r* = 0.06, *p* = 0.78; Figure 8E), nor did a line provide a good fit for the time bins near the ^18^F-FDG injections (*r* = 0.35, *p* = 0.40), as these data were best represented by a polynomial (Figure 8F).

**Table 4:** Changes in uptake in ROI relative to a reference region correlated with changes in behavioral activity across scan 2.

**Predict behavioral activity from**	**Coefficient r**		**Explained variance (%)**			
**ROI ratio**	**Linear**		**Quadratic**		**Cubic**	
	***n* = 23**	***n* = 8**	***n* = 23**	***n* = 8**	***n* = 23**	***n* = 8**
	**All time bins**	**Near injection**	**All time bins**	**Near injection**	**All time bins**	**Near injection**
**STR/**						
AMYG	−0.20 (0.35)	−0.43 (0.29)	4.34	22.73	6.43	52.39
AMYG+HIPP	–**0.39 (0.07)**	–**0.65 (0.08)**	16.44	44.25	17.16	44.33
HIPP	–**0.49 (0.02)**	−0.29 (0.49)	24.13	15.20	25.85	18.16
M	–**0.63 (0.001)**	−0.61 (0.11)	42.61	40.12	43.11	40.13
M+CB	−0.07 (0.76)	0.30 (0.47)	9.86	4.34	12.77	46.36
CB	0.27 (0.22)	0.61 (0.11)	7.25	61.42	11.49	62.00
AMYG+HIPP+M+CB	–**0.53 (0.009)**	−0.22 (0.61)	35.87	4.78	35.94	48.23
Average *R*^2^ for STR (%)	16.97	22.49	20.07	27.55	21.82	44.51
**M/**						
AMYG	**0.51 (0.01)**	0.34 (0.42)	26.48	14.06	26.61	18.47
AMYG+HIPP	**0.53 (0.009)**	0.48 (0.23)	29.25	24.38	29.41	25.30
AMYG+HIPP+STR	**0.57 (0.005)**	0.54 (0.17)	36.57	30.48	36.59	36.18
HIPP	**0.64 (0.001)**	**0.72 (0.05)**	45.82	57.37	45.85	90.23
STR+CB	**0.58 (0.004)**	**0.74 (0.04)**	34.18	82.90	37.82	96.08
CB	**0.53 (0.01)**	0.61 (0.11)	28.28	61.42	28.71	62.00
AMYG+HIPP+STR+CB	**0.59 (0.003)**	**0.77 (0.03)**	38.41	73.44	38.66	85.27
Average *R*^2^ forM (%)	32.01	38.12	34.14	49.15	34.81	59.08
**CB/**						
AMYG	0.17 (0.45)	0.37 (0.37)	7.13	46.83	12.06	59.33
AMYG+HIPP	0.13 (0.55)	0.37 (0.37)	6.35	46.83	14.70	59.33
HIPP	0.04 (0.86)	0.35 (0.40)	8.53	45.11	17.65	61.99
STR+M	0.03 (0.88)	0.35 (0.40)	14.21	45.11	16.84	61.99
AMYG+HIPP+STR+M	0.06 (0.78)	0.35 (0.40)	8.89	45.11	16.67	61.99
Average *R*^2^ for CB (%)	1.04	12.60	9.02	45.80	15.58	60.93
Average *R*^2^ for all ROIs (%)	16.59	24.40	21.08	40.83	24.07	54.84

*STR, striatum; AMYG, amygdala; HIPP, hippocampus;M,motor cortex; CB, cerebellum. AMYG+HIPP andM+CB, average of two regions; AMYG+HIPP+STR, average of three regions, etc. Results are based on rank-order correlations. Bold, p ≤ 0.08. Number in brackets, P-value*.

### PET-behavior studies in chickens usingmicroPET

**Figure 9:**
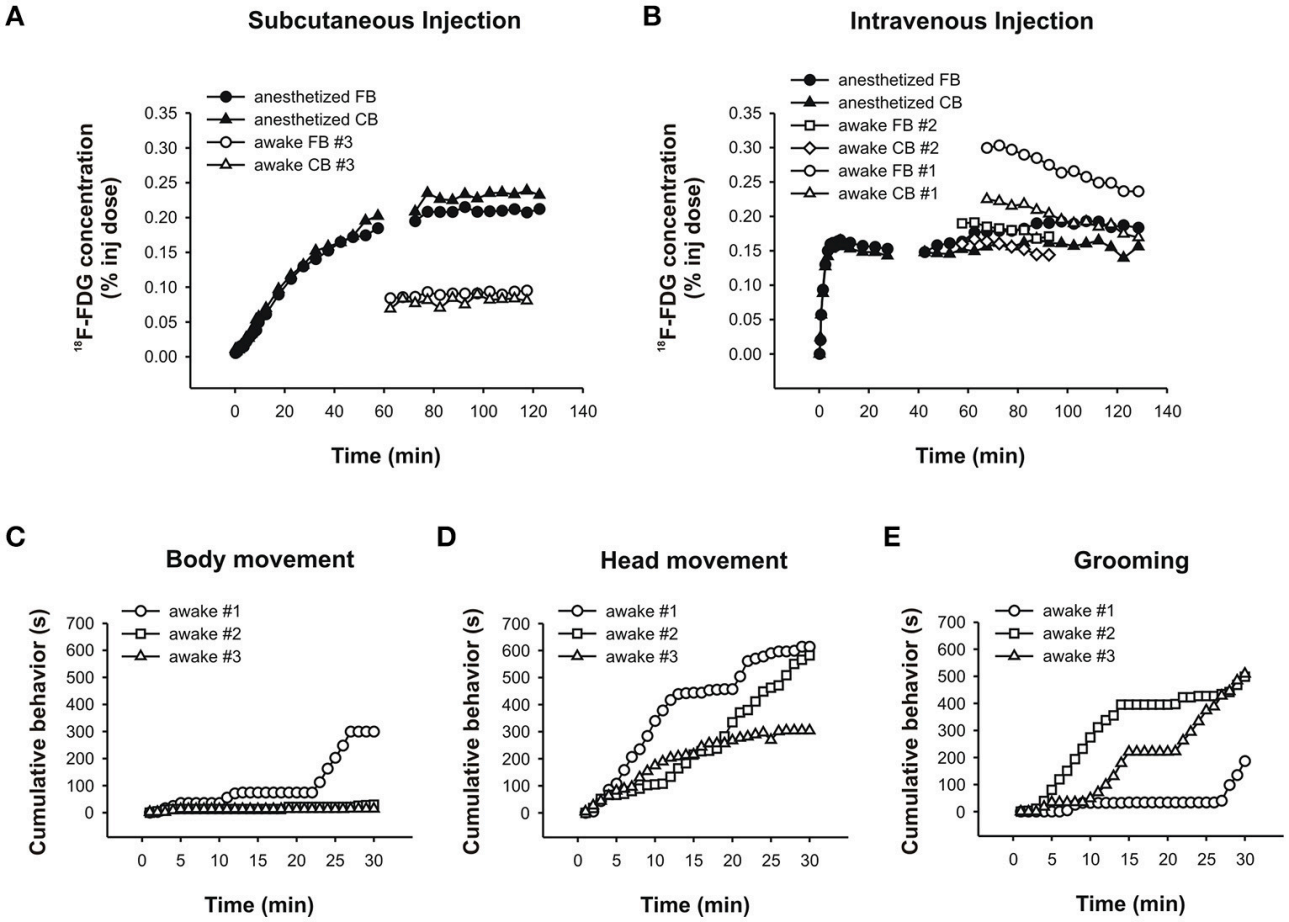
PET data and behavioral analyses for the chicken. Time-activity curves for the chicken following a **(A)** subcutaneous injection of ^18^F-FDG showing an anesthetized (black symbols) and an awake (white symbols) uptake study. Circles and triangles show uptake in the forebrain and cerebellum, respectively. **(B)** Intravenous injection of ^18^F-FDG showing an anesthetized (black symbols) and two awake (white symbols) uptake experiments. Circles and squares show uptake in the forebrain, and triangles and diamonds show uptake in the cerebellum. The time-activity curves for the anesthetized animal is shown in two parts due to subjectmotionmidway through the scan. Durations (s) of **(C)** bodymovement, **(D)** headmovement and **(E)** grooming were summed for each 1min time bin and cumulated. The rank order of chickens spending time engaging in each behavior changed when the initial 5–10min were considered versus the entire 30min of ^18^F-FDG uptake.

In the anesthetized chicken, a comparison of ^18^F-FDG uptake after s.c. and i.v. administration revealed that uptake time differed profoundly between these twomethods. When injected s.c., the tracer was taken up very slowly over a time period of about 80min (Figure 9A). After that, tracer uptake plateaued. By contrast, i.v. injections of ^18^F-FDG resulted in rapid uptake and a plateau after approximately 8min (Figure 9B).

In the “awake scans,” we didn't have the opportunity to capture the initial ^18^F-FDG uptake because the chickens were awake and active in a pen during uptake according to standard PET protocols (and were too large to be fitted with the RatCAP). Following uptake in the awake state, anesthesia and placement in the scanner, we found that tracer uptake after an s.c. injection of ^18^F-FDG had plateaued at 60min (Figure 9A, awake scan), somewhat faster than the data from the “anesthetized scan.” The time-activity curve was flat, indicating very little or slow tracermetabolism under this condition. By contrast, following i.v. injections of ^18^F-FDG the time-activity curves were decreasing by 60min of uptake time (Figure 9B, awake scans), indicating that not only uptake of ^18^F-FDG is faster after i.v. administration but also the tracer is removed earlier when the chicken is awake during the uptake. This information is relevant especially when behavioral assessments are to be correlated with ^18^F-FDG uptake.

We assessed bodymovement (Figure 9C), headmovement (Figure 9D), and grooming behavior (Figure 9E) during each scan. A comparison of the behavior that occurred by 8min (i.v. uptake time found in the present study) with the behavior that accumulated over 30min (typical uptake time allowed in PET-behavior studies), revealed important differences. Inmost cases, the quantity of behavior wasmore after 30min compared to 8min, consistent with the rat data.Moreover, the rank order of chickens performing a particular behavior changed with the assessed time period. For example, considering headmovement at 8min, chicken #1movedmore than #3 which, in turn,movedmore than #2. At 30min, #1movedmore than #2 which, in turn,movedmore than #3. Similarly, during the first 8min, chicken #2 groomed for longer durations than #3 and #1, but over the whole 30min, the rank order changed to #3 ≥ #2 > #1. Thus, consistent with the rat data, the chicken data suggest that confounding effects in brain-behavior studies can be reduced by accuratelymatching uptake time of the tracer with the occurrence of behavior for the same time period.

## Discussion

In summary, we found that in the awake, behaving ratmost of the ^18^F-FDG uptake occurred withinminutes and overlapped to a large extent with ^18^F-FDG data taken from longer uptake periods. By contrast, behavior which occurred withinminutes of the ^18^F-FDG infusion differedmarkedly from the behavior that occurred during later uptake periods. Accordingly, we found that changes in ^18^F-FDG uptake in STR,M, and CB relative to different reference regions significantly predicted changes in behavioral activity during the scan, if the time bins used for correlation were near the injection times of ^18^F-FDG. However, whenmorphine was also injected during the scan, which completely abolished behavioral activity for a prolonged period of time, a large proportion of the variance in behavioral activity was also explained by the uptake data from the entire scan. In chickens, uptake time differed profoundly as a function of injection route. Not only uptake of ^18^F-FDG was faster after i.v. compared to s.c. administration but also tracer removal occurred earlier in the awake, behaving chicken. Consistent with the rat data, the behavior of chickens changedmarkedly over the assessed time period, suggesting that random error could be reduced by accuratelymatching uptake time of ^18^F-FDG with the occurrence of behavior for the same time period.

In PET studies, behavior is correlated to a certain pattern of brain activity. ^18^F-FDG is a glucose analog so it is taken in by active neurons that need fuel. Hence, the behavior in questionmust occur during the period of tracer uptake because then the resulting images will show the active areas of the brain during the desired behavior. Standard PET scanning usually allows for 30–60min of ^18^F-FDG uptake inmammals before the induction of anesthesia and PET scanning. This uptake period is then used for the summation of all behavioral data and correlation with the PET data (Kornblum et al., 2000; Mirrione et al., 2007; Jang et al., 2009; Shackman et al., 2013; Thompson et al., 2014). It has not been previously possible tomeasure the actual uptake time of ^18^F-FDG in an awake, behaving rat because small animal PET scanners required complete stillness to image the animal withoutmotion artifacts. The invention of the RatCAP scanner that attaches to the animal's head has opened the door for simultaneous imaging and behavioral observation (Schulz et al., 2011; Gold et al., 2016). Using this device, the present study showed thatmost of the ^18^F-FDG uptake in an awake, behaving rat occurred within 5min or less. This insight is important to acknowledge if random error in brain-behavior correlations is to be reduced.

Firstly, when ^18^F-FDG data are treated in isolation, averagingmore rather than less data ismeaningful to reduce error related to photon counting statistics, also considering the relative consistency of the data across time. In this study, the data from the first 5min and first 25min overlapped to a large extent, ranging from ~60 to 80% for five different ROIs after the first ^18^F-FDG injection to over 90% following repeated injections. Thus, although the initial uptake ismost informative, the degree of uptake can be inferred from later time points due to the stability of the tracer once it is taken up. This stability is the result of a unique property of ^18^F-FDG which, unlike glucose, becomes trapped inside the cell as ^18^F-FDG-6-phosphate until decay of the isotope (Sokoloff et al., 1977; Gallagher et al., 1978). Thismetabolic trapping does not occur in all tissues equally but is highly evident in the brain and heart ofmammals (Gallagher et al., 1978).

By contrast, behavior is dynamic and variable across short periods of time. This short latency for change is a challenge for PET studies which collect relatively static data across longer periods of time but aim to reflect behavior in brain images in sensitive ways. In our example, the overlap in behavioral activity the rat showed in the first 5min and 25min after each ^18^F-FDG injection ranged from ~50 to 0%, indicating that the behavior can changemarkedly across short periods of time. Typically, behavioral activity will accumulate over time, andmore behavior will be displayed over longer compared to shorter periods of time, consistent with our data. On the other hand, rats are also known to become less active over time in response to an increase in familiarity of the environment (Berlyne, 1955, 1966). Consistent with this notion, we observed that under baseline conditions the rat's activity decreased across the first 5min bins following each ^18^F-FDG infusion, perhaps indicative of habituation. Other than this regularity, however, and the fact thatmorphine abolished all behavioral activity for about an hour of scan time, activity levels were variable.

We found that changes in ^18^F-FDG uptake were correlated with changes in behavioral activity during the scan, which suggests that our behavioral neuroimaging approach can be used with reversible (Schulz et al., 2011) and irreversible PET tracers. Specifically, we found that under baseline conditions changes in ^18^F-FDG uptake in STR,M, and CB, as defined by differences in average ^18^F-FDG concentrations between each of two successive 5min intervals, were predictive of changes in spontaneous behavioral activity, if the time bins captured the first ~10min following an ^18^F-FDG injection. This finding corroborates the conclusion thatmost of the information is contained wheremost of the uptake occurs and, thus, that uptake times of ^18^F-FDG shouldmatch the time periods used for behavioral analysis if random error in PET-behavior correlations is to be reduced. On the other hand, when the rat was injected withmorphine during the scan, a slightly different picture emerged. On average, the variance in behavioral activity was still best explained by the variance in uptake in STR,M, and CB when only the time bins near the ^18^F-FDG injections were included in the analyses. However, a large proportion of the variance in behavioral activity was also explained by the uptake data from the entire scan, though this was the case for STR andM, but not CB. Perhaps themorphine injection resulted in a separation of data categories, such as pre- and post-injection levels of behavioral activity and activity absenteeism in themiddle of the scan, which somehow produced the correlations artificially, although we do not have evidence for that. Perhaps the behavior was constant or predictable enough across time to producemeaningful correlations with the uptake data from the entire scan. Future RatCAP research will need to delineate the conditions that produce such differences between scans.

We chose to focus our analyses on STR,M, and CB because these regions are well known tomediatemotor functions (Albin et al., 1989; Berridge and Whishaw, 1992; Sanes and Donoghue, 2000; Doyon et al., 2003; Kishore et al., 2014), and in a previous study we found a correlation between changes in dopamine D2 receptor binding in STR and changes in behavioral activity in a rat wearing the RatCAP (Schulz et al., 2011). We have tested different ROIs, single, and combination ROIs, as reference regions to account for residual ^18^F-FDG from previous injections. While the choice of the amygdala and hippocampus were arbitrary, these regions are non-motor regions known tomediate emotional and contextualmemories (Morris et al., 1982; LeDoux, 1993), that we'd expect to have a relatively slow time course compared to the rapid dynamics ofmotor activity. On the other hand,motor functions, such as skill learning andmotor adaptation, also involvememory processes. Indeed, given some variability, wemostly found consistent results withmotor and non-motor ROIs as reference regions.

Interestingly, we found positive correlations between changes in STR uptake and behavioral activity under baseline conditions but negative correlations between the data sets whenmorphine was also administered during the scan. While ^18^F-FDG uptake in the STR is not specific to one cell type, the largemajority of cell bodies in this region are GABAergicmedium spiny neurons (MSNs), which are an important component of the striatal output pathways that organizemotor behavior (Kita and Kitai, 1988; Albin et al., 1989; Gerfen et al., 1990). These neurons contain dopamine receptors (Gerfen et al., 1990), whichmediate a net increase inmovement when activated by dopamine (Wichmann and DeLong, 2010). Importantly, these neurons also containmu-opioid receptors (MOR) to whichmorphine binds (Wang et al., 1996, 1997). In fact, a large number ofMORs are located on the dendrites ofMSNs that receive dopaminergic terminals from the substantia nigra (Wang et al., 1997), indicating thatmorphine couldmodulate the post-synaptic effects of dopamine receptor binding. A small number ofMORs are also located on axon terminals, but not dopaminergic terminals, which suggests that presynapticMORs on corticostriatal afferentsmodulate glutamatergic input into the striatum (Wang et al., 1997). Correlations between changes inM uptake and behavioral activity were positive throughout our study, which suggests that themodulation throughmorphine occurred downstream fromM but possibly via an effect of presynapticMORs on striatal neurons.MOR agonists given systemically increase dopamine release in STR, which was found to correlate with a biphasic effect on behavior at higher doses (Babbini and Davis, 1972; Di Chiara and Imperato, 1988). Consistent with these findings, we also observed thatmorphine completely suppressed behavioral activity for about an hour, which was followed by hyperactivity as indicated by the steep upward slope in the late part of the rat's cumulative response curve. While glucose uptake is not confined to specific subcellular domains such as cell bodies (Maher, 1995), our data suggest thatmorphinemodulated the functioning ofMSNs in STR, thereby impacting the associations between ^18^F-FDG uptake in STR and behavior.MORs appear to be absent in the rat cerebellum (Schadrack et al., 1999), so that themodulation from positive correlations with CB under baseline conditions to curvilinear correlations whenmorphine was injected during the scanmust be attributed to indirect effects on the cerebellum, possibly via descending pathways originating from themotor cortex (Kishore et al., 2014).

The dynamic-like injection protocol used with RatCAP in the present study allowed for a first demonstration of a link between changes in brain uptake of ^18^F-FDG and changes in behavior that occurred over the course of the PET scan. The presentmethod is a derivative of themethod we have used with ^11^C-raclopride in our earlier study (Schulz et al., 2011), with the difference that ^18^F-FDG is not a reversible tracer like ^11^C-raclopride. In both cases is the repeated administration of tracer necessary to be able to obtain neurochemicalmeasures that are capable of reflecting short-term behavioral changes. Such changes can only be assessed with a tool, such as RatCAP, which allows for the simultaneousmeasurement of brain and behavioral processes. While we chose to inject ^18^F-FDG every 30min in the present study, the relatively short uptake times suggest that shorter injection intervals could also be tested in the future to allow for an assessment of even shorter dynamics. It should be pointed out that the cabling and data acquisition systems that sit on top of the pendular support structure limit the animal'smovements. For example, rearings, a vertical form of exploratory behavior, and grooming behavior are not or very rarely observed. Thus, it will be important in future RatCAP research to increasemobilitymore and/or to construct behavioral paradigms that allow for greater variations in behavior.

The use of PET in avians is in its infancy, and has been used for veterinary diagnostics (Souza et al., 2010; Jones et al., 2013) and very few behavioral studies (Marzluff et al., 2012; Cross et al., 2013; Gold et al., 2016). The RatCAP provides an ideal way to study behaviors in birds, and while we were able to use it successfully in starlings (Gold et al., 2016), the size of the chickens and the presence of a waddle and comb impeded the use of the RatCAP. Inspired by the rapid uptake we observed in rats, and the comparability betweenmammals and avians, we were interested to explore uptake times in chickens.

We found several factors affecting uptake time in chickens. One of these factors was injection route. Here, anesthetized chickens were injected via s.c. and i.v. routes. The s.c. injection resulted in a long uptake time, approximately 80min. However, the i.v. injection resulted in approximately 8min of uptake time. This suggests that uptake period decreases in relation to increasing directness of the injection route to the circulatory system.Most previous avian PET studies have used i.v. injection routes either through the jugular (Souza et al., 2010) or basilica (Jones et al., 2013) veins, though Marzluff et al. (2012) and Cross et al. (2013) used an i.p. injection in their behavioral studies. Even though birds have a peritoneal cavity, injection into this cavity is not recommended due to the presence of air sacs scattered throughout the abdomen (Fowler, 2007; Fair et al., 2010).Muscles provide a secure injection route formost drugs in birds without endangering the air sac system. By contrast, i.m., and s.c. injections are not usually used in rodent PET studies. However, one study found that ^18^F-FDG injected s.c. resulted in low levels of uptake in lymph nodes (Wahl et al., 1990). In rats, i.v. injections are preferred although i.p. injections are also sometimes used. One study in anesthetized rats demonstrated that ^18^F-FDG concentrations peaked after 30min using an i.p. route, but peaked in less than 10min using an i.v. route (Schiffer et al., 2007). Researchers couldmake use of this knowledge by choosing an injection route that accommodates behaviors of different durations. This would insure that the behavior is fully captured during the uptake time.

We havemeasured three behaviors in chickens while in a pen—bodymovement, headmovement, and grooming. Overall, the activity levels were higher if cumulated over longer than shorter periods of time, consistent with the rat data. Importantly, the chickens differed in the durations that they displayed each behavior across time, at times converging and at times diverging in the amount of performance. In the case of headmovements and grooming behavior, the rank order of chickens performing each behavior was different for the first 8min of ^18^F-FDG uptake and the first 30min. Thus, depending on the time period used to assess the behavior of interest for correlation with the PET data, different results would be obtained. Future avian researchmight benefit from comparing shorter with longer time periods used for behavioral assessments that are adjusted to uptake time and, thus, injection route for correlation with the PET data.

While we had considerable variability in the protocols of our chicken studies, the differences in the fasting protocols were unlikely to have affected glucose uptake and, thus, ^18^F-FDG concentrations in the tissues. Birds, including chickens, show a remarkable stability in blood glucose levels even after days of fasting (Belo et al., 1976; Stevens, 1996). Less is known about the influence of different anesthetics on PET imaging in birds. The few studies that have been conducted to date, have used isoflurane anesthesia (Souza et al., 2010; Marzluff et al., 2012; Cross et al., 2013; Jones et al., 2013). We have also tested isoflurane in the chickens first, but found it to be unsuitable due to respiratory ailments even after injection of glycopyrrolate which is commonly used to reduce salivation. On the other hand, the i.m. administration of a ketamine/xylazinemixture presented a workable solution. The impact of the use of different anesthetics as well as other factors, such as age and sex, on the uptake of ^18^F-FDG in birds remains to be illuminated in future studies.

In the few chickens we have studied, we observed an increase in ^18^F-FDG uptake over time or, depending on the ROI, stable concentrations in anesthetized chickens but amarked decrease in ^18^F-FDG concentration following i.v. infusions in the awake state. An explanation of these observations will require some speculation. In birds, unlikemammals, the cellmembrane ismuch less permeable to glucose, and small amounts of glucose are taken up relative to high extracellular concentrations. The uptake increases, however, in the presence of nucleosides like adenosine, at least in red blood cells (Stevens, 1996). Adenosine hasmany functions, but its increase in the brain is associated with lowmetabolic states, such as sleepiness and anesthesia (Huston et al., 1996; Gettys et al., 2013). Thus, perhaps the increase in ^18^F-FDG uptake in anesthetized chickens was due to an accumulation of adenosine or other carriers in tissue that were not present in the awake state. Nevertheless, ^18^F-FDG concentrations in the chickens were fairly stable following the initial uptake. By contrast, in the avian *Sturnus vulgaris* (Common starlings) we have observed a very fast washout of ^18^F-FDG that we prevented by administration of insulin (Gold et al., 2016). Marzluff et al. (2012) also recorded a very fast washout of ^18^F-FDG in crows.

Imaging birds with PET scanning began only recently outside of the veterinary field, primarily to provide a healthy comparison to sick animals (Souza et al., 2010; Jones et al., 2013). Of themany birds that are kept as pets or in zoos, only Hispaniolan parrots (Souza et al., 2010) and bald eagles (Jones et al., 2013) have been examined with ^18^F-FDG. These studies analyzed tracer uptake in a variety of organs, but did not study how differences in injection route changes uptake rates. One other study evaluated the activity in the brain of crows while viewing different human faces (Marzluff et al., 2012). More recently, we have studied starlings and found that the uptake time following an i.v. injection of ^18^F-FDG was 3–4min in the awake state (Gold et al., 2016). The present study expands the taxonomic sampling of birds using PET. These data can be used as a comparison to sick animals or as a baseline to continue behavioral studies in avians.

## Conclusion

In conclusion, the rapid uptake times of ^18^F-FDG both in the awake, behaving rat and the anesthetized chicken together with the rapid dynamics of rat and chicken behavior suggest that uptake timesmatched accurately with behavior for the same time periods will reduce random error in brain-behavior correlations. Furthermore, we have explored a new, dynamic-like ^18^F-FDG injection protocol for use with the RatCAP scanner, which allowed us to repeatedly assess uptake during behavior across a 120min scan. Using thismethod, we showed thatmost information in an ^18^F-FDG scan is located wheremost of the uptake occurs, which, in turn, predicted changes in behavioral activity. Drug interventions or conditions thatmake behaviormore predictablemight increase the window under which PET-behavior correlations become observable. Future research will need to resolve the question whether group averages need to be the goal of RatCAP research or whether the statistics that we generate from time series in individual animals are a promising avenue thatmay help personalizedmedicine. It will also be of interest to analyze other behaviors for correlation with ^18^F-FDG. This will require properly designed paradigms that increasemobility and are suitable for use with RatCAP tomography. While avian PET scanning is in its infancy, our previous work with starlings (Gold et al., 2016) and our current work with chickens provides impetus for comparative studies that in the long run could help determine brain function.

## Author contributions

MG, MN, PV, and DS designed the experiments. MG, MB, and DS ran the analyses and interpreted the data. MG, MN, PV, and DS wrote the manuscript. MG, MN, MB, PV, and DS approved of the manuscript.

### Conflict of interest statement

The authors declare that the research was conducted in the absence of any commercial or financial relationships that could be construed as a potential conflict of interest.
